# Metabolomic Analysis in Brain Research: Opportunities and Challenges

**DOI:** 10.3389/fphys.2016.00183

**Published:** 2016-05-24

**Authors:** Catherine G. Vasilopoulou, Marigoula Margarity, Maria I. Klapa

**Affiliations:** ^1^Metabolic Engineering and Systems Biology Laboratory, Institute of Chemical Engineering Sciences, Foundation for Research and Technology—Hellas (FORTH/ICE-HT)Patras, Greece; ^2^Human and Animal Physiology Laboratory, Department of Biology, University of PatrasPatras, Greece; ^3^Departments of Chemical and Biomolecular Engineering and Bioengineering, University of MarylandCollege Park, MD, USA

**Keywords:** CNS metabolomics, systems biology, systems medicine, neurophysiology, network medicine, metabolic network analysis, metabolomic data standardization

## Abstract

Metabolism being a fundamental part of molecular physiology, elucidating the structure and regulation of metabolic pathways is crucial for obtaining a comprehensive perspective of cellular function and understanding the underlying mechanisms of its dysfunction(s). Therefore, quantifying an accurate metabolic network activity map under various physiological conditions is among the major objectives of systems biology in the context of many biological applications. Especially for CNS, metabolic network activity analysis can substantially enhance our knowledge about the complex structure of the mammalian brain and the mechanisms of neurological disorders, leading to the design of effective therapeutic treatments. Metabolomics has emerged as the high-throughput quantitative analysis of the concentration profile of small molecular weight metabolites, which act as reactants and products in metabolic reactions and as regulatory molecules of proteins participating in many biological processes. Thus, the metabolic profile provides a metabolic activity fingerprint, through the simultaneous analysis of tens to hundreds of molecules of pathophysiological and pharmacological interest. The application of metabolomics is at its standardization phase in general, and the challenges for paving a standardized procedure are even more pronounced in brain studies. In this review, we support the value of metabolomics in brain research. Moreover, we demonstrate the challenges of designing and setting up a reliable brain metabolomic study, which, among other parameters, has to take into consideration the sex differentiation and the complexity of brain physiology manifested in its regional variation. We finally propose ways to overcome these challenges and design a study that produces reproducible and consistent results.

## The systems biology revolution and its impact on brain research

The new technologies for high-throughput biomolecular analysis (aka “omics”) enabled the simultaneous quantification of tens to thousands of molecules at various levels of cellular function. The comprehensive perspective of molecular physiology that can now be obtained renders obsolete the “conventional” reductionist approach (Ge et al., 2003; Vidal, 2009), as biological systems can be viewed as networks of interacting genes and gene products (Vidal and Cusick, 2011).

In the case of central nervous system (CNS) research in particular, network and systems biology greatly enhanced the available analytical toolbox, when considering the highly complex, multi-scale in space and time, structure of the mammalian brain (Geschwind and Konopka, 2009; Bassett and Gazzaniga, 2011). For a comprehensive understanding of mammalian brain architecture and function, CNS activity has to be viewed as the coordinated interaction of the biomolecular networks of the various brain regions. The functional role and properties of brain regions are synergistic and complimentary, but not linearly dependent, requiring thus systems biology approaches for their elucidation and interpretation. The high-throughput quantitative nature of omics studies shifts brain research toward knowledge-based systemic investigations (Reddy et al., 2013), distinguishing them from the mainly hypothesis-driven currently performed in the majority of neurobiology laboratories. The systemic studies can lead to a comprehensive mapping of mammalian brain function with respect to neural connections (“connectomics,” e.g., Lichtman et al., 2014) and molecular fingerprinting [transciptomics, proteomics, metabolomics (Geschwind and Konopka, 2009)]. These results are expected to advance the *in vivo* neuroimaging and functional analysis techniques, like PET, MRI, optogenetics (de Celis Alonso et al., 2015; Jarvis and Schultz, 2015; Lu and Yuan, 2015), toward the development of sensitive and accurate diagnostic tools and the design of personalized therapeutic treatments.

## The emerging role of metabolomics

Metabolomics is the most recently introduced among all omics, with a very rapid growth in the last years. It refers to the high-throughput analysis of the metabolic network state, through the simultaneous quantification of the concentrations of free low molecular weight metabolites, i.e., the metabolic profile. Since free metabolite concentrations affect and are affected by the metabolic reaction rates (or fluxes), the metabolic profile is a metabolic fingerprint, providing a perspective of the *in vivo* enzymatic activity, which cannot be obtained by transcriptomics or proteomics (Hollywood et al., 2006; Kanani et al., 2008; Patti et al., 2012). Metabolomics can be readily applied to biological systems under transient physiological conditions, does not require extensive knowledge of the metabolic network structure and uses classical analytical chemistry techniques (Kanani et al., 2008). The metabolomic data can contribute to the reconstruction of the active metabolic network, information which in the case of mammalian brain research can be incorporated to the comprehensive neural connectome (Sporns, 2011; Ivanisevic and Siuzdak, 2015). Despite its significance, the broad deployment of metabolomics in systems medicine in general and brain research in particular, is currently hindered by the lack of standardized methodologies ensuring accurate and reproducible results.

In this technical context, few large-scale metabolomic studies of CNS disorders in human have been reported so far, having, however, already demonstrated the value of high-throughput vs. small-scale metabolic investigations. They focus mostly on the untargeted profiling of biological fluids, blood plasma or serum, and cerebrospinal fluid (CSF) (Holmes et al., 2006; Kaddurah-Daouk et al., 2012; Yang et al., 2013; Yoshimi et al., 2016) and a limited number, on the metabolic fingerprinting of post-mortem human brain (Prabakaran et al., 2004; Chan et al., 2011; Graham et al., 2013; Jové et al., 2014), mainly for the investigation of neurodegenerative and psychiatric disorders. For a comprehensive profiling of brain tissue avoiding any complications associated with post-mortem studies, animal models have been used (e.g., Salek et al., 2010; Constantinou et al., 2011; Davidovic et al., 2011; Chen et al., 2015; González Domínguez et al., 2015). These studies provide a holistic perspective of the metabolic alterations underlying brain dysfunction, furthering our understanding of the molecular basis of disorders like schizophrenia, bipolar disorder, depression, or Alzheimer's disease (AD), opening new research directions and leading the way toward the detection of specific biomarkers for the development of personalized diagnostic tools and treatments (Guest et al., 2015).

## Standardizing brain metabolomics in systems biology research

**Figure 1:**
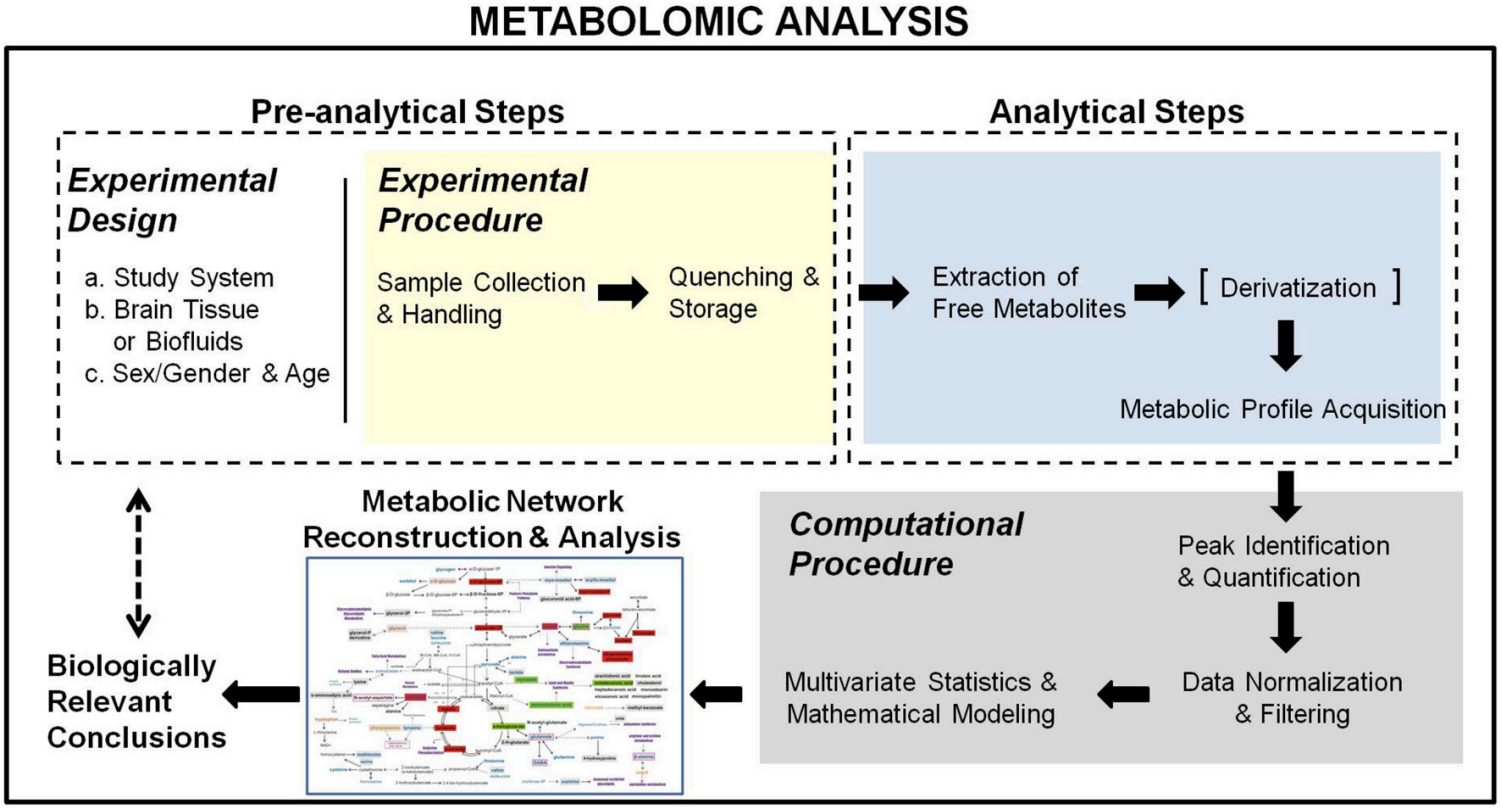
**Schematical representation of the metabolomic analysis workflow**. It comprises a pre-analytical, an analytical, and a computational section, starting from an educated experimental design toward the reconstruction of the metabolic network activity map for the extraction of biologically relevant conclusions.

Metabolomics in systems biology aims at using the metabolic profiles to elucidate the dynamics of metabolic pathways and reveal molecular mechanisms of disorders. This objective is even more prominent in the context of integrated analyses with other omics (Martins-de-Souza, 2014). Therefore, metabolomics should not be viewed as a mere chemometric methodology employing high-tech analytical chemistry techniques, but as a multi-step biomolecular analysis, correspondent at the metabolic level of the transcriptomic and proteomic profiling. In this context, metabolomics comprises both experimental (pre-analytical and analytical) and computational parts (Figure 1). It starts from the educated selection of the biological system and study group along with the appropriate experimental design to ensure accurate and sensitive metabolic activity monitoring, based on the investigated biological question(s), the handling and collection constraints of the utilized biological system and the requirements of the analytical technique(s) used for the metabolic profile acquisition. It ends with the reconstruction of the relevant metabolic activity network, used to validate and interpret the acquired metabolomic data with respect to the examined biological problem. Below, we describe the metabolomic analysis steps, pointing out issues in their design and/or execution to be considered when applied in brain research and proposing ways to address these concerns, toward standardized analyses with validated performance.

### Pre-analytical section

#### Experimental design

In human neurophysiology investigations, metabolomic analyses have to rely on biofluids, mainly blood and CSF. However, given the blood-brain barrier (BBB), there are serious doubts whether sensitive biomarkers of neurodegenerative disorders should be sought in the blood composition (Trushina et al., 2013). CSF reflects better the brain physiology, as shown for depression and AD, respectively, by the metabolomics studies of Kaddurah-Daouk et al. (2012) and Lista et al. (2014). However, due to the invasiveness of the collection procedure, most available samples are from patients of brain disorders, who have to undertake it as part of their monitoring or treatment. Thus, patient profiles of a disorder are often used as reference in the analysis of another, contributing thus to a high variation between studies. Addressing this issue requires educated experimental designs with a large number of patients, if available, and sophisticated statistical methods. Brain tissue analysis is based primarily on animal models. Post-mortem human tissues are in low availability and vary with respect to patients' characteristics, disease state, therapeutic treatment, cause of death, collection, and handling procedure, differences that can affect the acquired metabolic profiles (Samarasekera et al., 2013). On the other hand, animal model studies depend on the model accuracy in simulating human pathophysiology (Suvorov and Takser, 2008; McGonigle and Ruggeri, 2014). Regarding metabolism in particular, a disorder may cause opposite effects in lab rodents compared to humans (Panzoldo et al., 2011; Blekhman et al., 2014; Martens, 2015). Brain regional variation should also be considered, as a comprehensive perspective of brain pathophysiology can be obtained from the integrated analysis of multiple brain regions (e.g., Salek et al., 2010; Ivanisevic et al., 2014).

**Figure 2:**
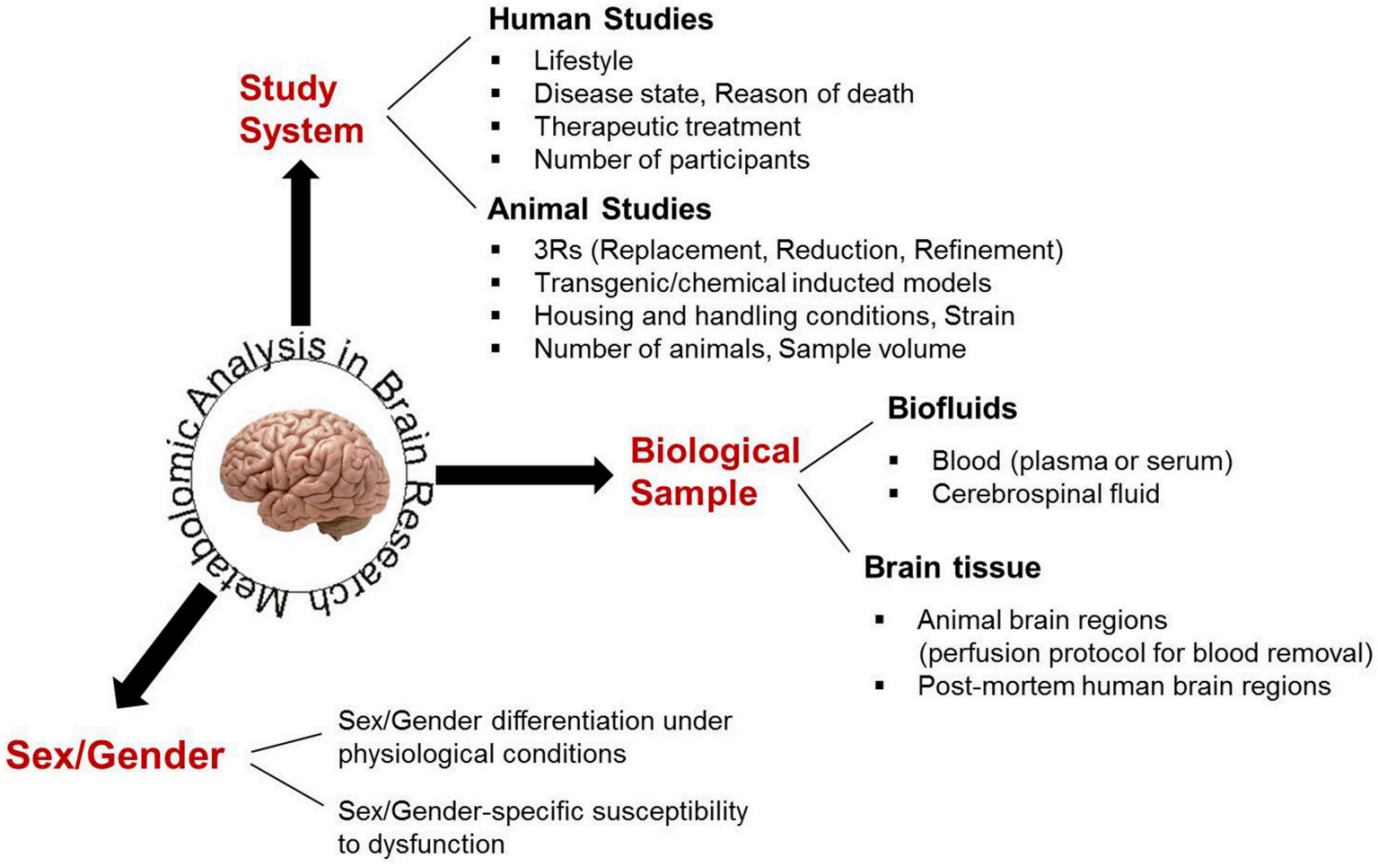
**The main biological parameters that have to be considered for an educated experimental design in brain metabolomics**.

In human investigations, study groups should be selected to be matching with respect to gender, age, and other demographic characteristics. Accuracy and reproducibility between metabolomic studies in animals require well-controlled housing and handling conditions, including standardized food tailored to the study objective(s) (Rathod et al., 2014; Selfridge et al., 2014) and minimization of any stress situations (Liu et al., 2013; Heinla et al., 2014) that may affect brain metabolic physiology. Educated experimental designs allow for mainly the investigated parameter(s) to be affecting the acquired results, decreasing the impact of biological variation. Thus, they support the use of few animals, adhering to the “3R” (Replacement, Reduction, Refinement) regulations for animal studies (Graham and Prescott, 2015). Brain studies cannot be limited to the analysis of one sex/gender or to mixed samples from both sexes, as the effect of a particular pathophysiology or the impact of a therapeutic treatment may greatly differ between sexes (Cahill, 2006; Beery and Zucker, 2011; Miller, 2014), shown also in the metabolomics study of Zhang et al. (2009). Finally, the experimental design of a CNS metabolomic analysis should take into consideration the requirements of the analytical technique used for profile acquisition, regarding the amount of biofluid or the size of brain tissue required for an accurate and reproducible performance. In light of the available sample number, pooling may be necessary, despite preventing personalized profiling (Schmidt et al., 2009; Chinopoulos et al., 2011). All parameters to be considered in the experimental design of brain metabolomic studies are schematically shown in Figure 2.

#### Sample collection and handling

The sample collection and handling is a key step in a metabolomic analysis as it has to minimally affect the metabolic physiology of brain tissue and minimize metabolite loss and/or significant alterations in metabolite concentrations in the case of biofluids, before the enzymatic quenching. International directives for collection, handling, storage, and transport for samples to be used in omic analyses are currently under development to enable the generation of extensive and comparable biobanks for omic studies. In all cases, it is important that the collection and handling procedure has the shortest duration possible and—if possible—carried out at 4°C. For these reasons, in blood metabolomics, plasma has been preferred over serum (Liu et al., 2010; Yu et al., 2011). Moreover, the anticoagulant cannot be a metabolic intermediate, should minimally interact with the blood components and its measurement could be easily separated from the metabolic profile. Thus, EDTA has been preferred over heparin for metabolomic and proteomic analyses (Hebels et al., 2013). CSF collection requires highly trained clinicians as the procedure is risky and painful (Quinones and Kaddurah-Daouk, 2009). In post-mortem tissue analyses, metabolomic data normalization should take into consideration the time after death, the tissue removal method and duration, the sample freezing method and storage duration (Samarasekera et al., 2013).

In brain tissue analyses of animal models, there is still a debate regarding the use of perfusion protocols. As the head can be isolated from the rest of the body, most reported neurochemistry studies are based on non-perfused tissues. Perfusion introduces an additional perturbation potentially affecting the metabolic physiology of the animal in general and brain tissue in particular. However, the application of a perfusion protocol is imperative to separate the brain tissue profile from the blood metabolite composition, while it cannot be avoided when parallel multi-organ studies are concerned. PBS or isotonic saline have been used as perfusion agents. Further studies comparing the effect of various perfusion protocols on brain metabolomics are required. In the same context, the use and method of anesthesia are still debatable in animal brain studies. Following the anesthesia procedures considered mildest for human should be the most appropriate measure for metabolomic analyses, although definite answers for this subject require specialized investigations (Overmyer et al., 2015). For example, using dry ice for anesthesia is not appropriate for metabolomic studies as carbon dioxide may perturb the animal metabolism.

### Analytical section

#### Free metabolite extraction

There is no extraction method for the entire metabolome. The most commonly used solvents are methanol(/water) for polar metabolite extraction and chloroform for lipidomic analyses (Kanani et al., 2008). Various other extraction solvents exist and protocols should be appropriately selected based on the study objective.

#### Selection of the analytical technique

Metabolomics uses classical analytical chemistry techniques, like NMR and mass spectrometry (MS) (Spagou et al., 2011). NMR is of lower throughput and sensitivity than MS, but all measured molecules are identified. MS is preferred for untargeted metabolomics, but there are many unidentified peaks. Between gas chromatography (GC)-MS and liquid chromatography (LC)-MS, the former is more sensitive, of higher metabolite resolution and referring to more populated peak databases. However, it is limited to compounds of molecular weight lower than 650–1000 daltons and requires metabolite derivatization, with any associated biases that need appropriate experimental design and data normalization (Kanani and Klapa, 2007; Kanani et al., 2008). If possible, integration of analytical techniques should be preferred because the technological platforms provide complementary information, as supported by the combined GC-MS and UPLC-MS analysis of González Domínguez et al. (2014).

### Computational section

#### Peak identification and quantification

Peak identification and quantification is carried out according to the analytical technique used. The analysis of NMR spectra is more standardized compared to that of the MS-reconstructed chromatograms of untargeted metabolomics in particular. Software tools for automated feature identification and quantification are used. In LC-MS, in particular, some researchers use the entire feature profile for multivariate statistical analysis in a purely data-driven approach (Meinhardt et al., 2015). However, the “metabolite-centric” approach, in which a marker ion is selected to represent and quantify each metabolite derivative, has been considered more accurate. This method is “knowledge-driven,” filtering out any biologically irrelevant chromatographic artifacts and avoiding the inclusion of mathematical biases in subsequent analysis, originated from the multiple linearly dependent peaks/features belonging to the same metabolite (Kanani et al., 2008; Allwood et al., 2009).

#### Data validation, normalization, and filtering

A significant part of the computational analysis of untargeted omic profiles is data validation and normalization (Quackenbush, 2001; Kanani et al., 2008). At this step, quality control (QC) measures are used to evaluate whether all acquired data refer to the same experimental procedure conditions and are not subject to systematic experimental biases that can skew their biologically relevant differences. To this end, QC reference samples are run before, during and after the acquisition of the metabolic profiles of an experimental batch (Gika et al., 2016), internal standards are used and other specialized normalization methods [e.g., for GC-MS derivatization biases (Kanani et al., 2008)] are applied to ensure comparability between the analyzed profiles. After normalization, data that are not consistently detected, have a low signal to noise ratio, are technical artifacts or are suspect of experimental biases for which they cannot be appropriately corrected, are filtered out of further analysis. Standardized QC, normalization and filtering methods can promote validated performance among laboratories and assist in the formation of integrated metabolomic databases for meta-analysis (Steinbeck et al., 2012; Wishart et al., 2013).

#### Multivariate statistical analysis–metabolic network reconstruction

Metabolomic data could be analyzed using multivariate statistical analysis methods for the extraction of correlations between the various profiles or metabolites, in accordance with other omics. However, metabolite concentrations may differ over a wide range of orders of magnitude, so it may be preferable for standardized values (z-scores) to be used. Furthermore, as the metabolite concentrations are related through the metabolic pathway network structure and regulation, interpreting the metabolomic data in the context of the known metabolic network greatly enhances their information content, assisting both in the validation of the statistical analysis results and in the extraction of biologically relevant conclusions. A major advantage of metabolomics over other omics is that there is a larger knowledge of the metabolic compared to other biomolecular networks. Related tools predicting metabolic pathway activity from metabolomic data include pathway activity prediction profiling—PAPi (Aggio et al., 2010) and metabolite set enrichment analysis—MSEA (Xia and Wishart, 2010).

Genome-scale reconstructed metabolic networks are available for human (Duarte et al., 2007) and mouse (Sigurdsson et al., 2010). The metabolic network reconstruction for specific organs can be based on the genome-scale reconstructed network of the particular species and relevant information from metabolic databases [e.g., KEGG (Kanehisa et al., 2012); MetaCyc (Caspi et al., 2014)], literature and available metabolomic data. In this way, using mainly transcriptomic and proteomic data, genome-scale metabolic models of human brain tissues have been developed (Hao et al., 2012; Wang et al., 2012). Accordingly, we have reconstructed a concise version of the primary metabolism network of the mouse brain and used it for metabolomic data interpretation (Constantinou et al., 2011). However, questions about the potential reversibility of certain reactions and the ability of the brain to synthesize certain metabolites vs. obtaining them only through BBB remain open, to be answered by specialized metabolic investigations preferably with the use of labeled compounds and CSF metabolomic data. For the metabolomic analysis of biofluids, there is a need for reconstructing inter-organ metabolic networks that connect the biofluid composition with the metabolic activity of the tissues that contribute to it. This is a challenging task as the relevant knowledge is not extensive. Combining information from human metabolism and endocrinology textbooks and literature, we reconstructed a network connecting the blood plasma metabolic profile to the central carbon metabolism of liver, adipose, muscle, and other tissues (Gkourogianni et al., 2014). To the best of our knowledge, there is no CSF metabolomic analysis connecting this data with the brain metabolic network.

## Conclusions and future directions

In the systems biology era, there is a shift from the reductionist approach toward the use of high-throughput biomolecular analyses and the interpretation of high-dimensional biomolecular profiles in the context of networks of genes and gene products. Metabolomics emerges as the newest omic analysis that provides a metabolic physiology fingerprint that can complement the transcriptional and the protein profiles, while providing additional information about the *in vivo* enzymatic activity and regulation. In brain research, the revolutionary perspective of systems biology triggers the combination between molecular biology and neurophysiology toward a new challenging research field that could be named as molecular systems neurophysiology. The application of metabolomics to the study of CNS physiology and pathophysiology will further our understanding of the CNS metabolic complexity, expected to provide important insight about the onset, progression, and treatment of multifactorial neurodegenerative and psychiatric diseases. However, for the successful deployment of metabolomics in brain research, issues related to pre-analytical and analytical steps along with the standardization of metabolomic data validation and handling have to be addressed to support its vast utilization as a major systems biology tool. Accurate reconstruction of the brain metabolic network, specialized for each brain region, is required. Moreover, systematic multi-organ studies in which the brain physiology changes are directly compared with alterations in peripheral tissues could provide a better understanding of the brain activity at the body level.

## Author contributions

MIK conceived, initiated, and formulated this perspective. MIK provided her systems biology experience and supervised CGV's research of the literature and systems neurophysiology metabolomic experiments and analyses that contributed to this perspective. MM contributed her expertise on neurophysiology research and practice. CGV and MIK drafted the manuscript, MM edited, and MIK finalized the manuscript. All authors have read and approved the final manuscript.

### Conflict of interest statement

The authors declare that the research was conducted in the absence of any commercial or financial relationships that could be construed as a potential conflict of interest.
